# Supplementary material for: The relationship between a plant-based diet and mental health: Evidence from a cross-sectional multicentric community trial (LIPOKAP study)
Source: PLoS One. 2023 May 31;18(5):e0284446. doi: 10.1371/journal.pone.0284446 (PMC10231825; doi:10.1371/journal.pone.0284446)
Supplement: S1 Table — (DOCX) [file pone.0284446.s001.docx]

**Supplementary Table 1-** General characteristics of participants across the quartiles of PDI, hPDI, uPDI scores, stratified by depression and anxiety status.

|  | Non Depression | Depression | P value | Non anxiety | Anxiety | P value |
| --- | --- | --- | --- | --- | --- | --- |
| Age (y)^a^ | 39.1±13.6 | 42.2±14.2 | <0.0001 | 39.1±13.8 | 41.8±13.6 | <0.0001 |
| Physical activity^b^ | 3116.21±3930.0 | 3413.0±4648.0 | 0.51 | 3146.2±3947.4 | 3273.6±4533.5 | 0.900 |
| Male (%)^c^ | 51.4 | 32.9 | <0.0001 | 52.8 | 29.5 | <0.0001 |
| Married (%)^c^ | 83.4 | 79.6 | <0.0001 | 82.8 | 82.3 | <0.0001 |
| Education year (%)^c^ |  |  | <0.0001 |  |  | <0.0001 |
| 0-5y | 20.0 | 36.2 |  | 19.7 | 35.5 |  |
| 5-12y | 45.6 | 46.4 |  | 45.6 | 46.5 |  |
| >12y | 34.4 | 17.3 |  | 34.7 | 18.0 |  |
| Current smoker (%)^c^ | 11.5 | 11.7 | 0.89 | 11.9 | 10.1 | 0.300 |

PDI, overall plant-based diet index; hPDI, healthful plant-based diet index; uPDI, unhealthful plant-based diet index.

Values are mean±SD for continuous variables and percentage for dichotomous variables.

^a^ p-value obtained based on robust Brown–Forsyth test.

^b^ p-value obtained based on Kruskal–Wallis test.

^c^ p-value obtained based on Chi-square test.
