# Supplementary material for: The relationship between a plant-based diet and mental health: Evidence from a cross-sectional multicentric community trial (LIPOKAP study)
Source: PLoS One. 2023 May 31;18(5):e0284446. doi: 10.1371/journal.pone.0284446 (PMC10231825; doi:10.1371/journal.pone.0284446)
Supplement: S2 Table — (DOCX) [file pone.0284446.s002.docx]

**Supplementary Table 2** Dietary intakes of study participants across the quartiles of PDI, hPDI, uPDI scores, stratified by depression and anxiety status.

|  | Non Depression | Depression | P value^a^ | Non anxiety | Anxiety | P value^a^ |
| --- | --- | --- | --- | --- | --- | --- |
| Energy (kcal/d) | 2314.93±762.64 | 2209.94±873.07 | <0.0001 | 2304.02±777.03 | 2260.29±818.24 | 0.091 |
| Carbohydrate (g/day) | 285.59±102.55 | 271.65±107.52 | 0.001 | 284.36±104.29 | 277.55±101.19 | 0.077 |
| Protein (g/day) | 95.97±34.84 | 88.52±36.69 | <0.0001 | 95.41±35.48 | 91.28±34.54 | 0.022 |
| Fat(g/day) | 92.67±36.79 | 89.31±43.28 | 0.007 | 92.39±37.30 | 90.65±41.08 | 0.170 |
| Fiber (g/d) | 22.86±8.38 | 21.68±8.60 | 0.003 | 22.74±8.45 | 22.22±8.34 | 0.128 |
| SFA (g/day) | 33.37±15.42 | 33.59±18.24 | 0.064 | 33.21±15.61 | 34.14±17.34 | 0.989 |
| MUFA (g/day) | 28.26±12.16 | 27.35±14.25 | 0.010 | 28.24±12.36 | 27.51±13.42 | 0.105 |
| PUFA (g/day) | 26.59±13.20 | 25.20±14.11 | 0.007 | 26.45±13.25 | 25.88±13.89 | 0.201 |
| Healthy vegetables (g/day) | 294.81±130.92 | 295.13±149.31 | 0.429 | 296.1±132.66 | 290.31±141.66 | 0.252 |
| Fruits (g/day) | 251.99±151.39 | 228.14±159.03 | 0.001 | 248.86±149.55 | 242.01±165.78 | 0.063 |
| Unhealthy vegetables (g/day) | 19.17±24.82 | 18.07±25.99 | 0.028 | 19.04±24.81 | 18.66±25.94 | 0.183 |
| Legumes (g/day) | 27.78±29.09 | 21.96±22.58 | 0.001 | 27.21±28.42 | 24.62±26.55 | 0.082 |
| Nuts (g/day) | 26.20±28.59 | 22.18±28.21 | <0.0001 | 25.59±28.07 | 24.80±30.30 | 0.068 |
| Refined grains (g/day) | 237.00±150.51 | 223.38±142.14 | 0.150 | 238.23±152.47 | 220.15±134.66 | 0.117 |
| Whole grains (g/day) | 90.26±96.89 | 88.18±100.56 | 0.204 | 89.01±96.55 | 93.00±101.34 | 0.921 |
| Meat (g/day) | 74.28±44.82 | 70.15±49.75 | 0.014 | 74.58±45.48 | 69.45±47.09 | 0.013 |
| Fish & sea food (g/day) | 16.27±16.79 | 11.69±13.97 | <0.0001 | 16.33±16.95 | 11.89±13.52 | <0.0001 |
| Dairy (g/day) | 356.62±235.09 | 328.06±237.89 | 0.004 | 353.05±234.60 | 344.02±240.34 | 0.259 |
| Fast food (g/day) | 13.26±19.91 | 14.61±27.23 | 0.033 | 13.52±20.57 | 13.49±24.69 | 0.021 |
| Sweet dessert (g/day) | 10.04±13.19 | 11.05±14.17 | 0.523 | 10.00±13.03 | 11.10±14.61 | 0.500 |
| Sweet drink (g/day) | 35.82±51.91 | 38.24±58.80 | 0.524 | 36.97±53.09 | 33.75±54.06 | 0.013 |

PDI, overall plant-based diet index; hPDI, healthful plant-based diet index; uPDI, unhealthful plant-based diet index.

SFA: saturated fatty acid; PUFA: polyunsaturated fatty acid; MUFA; monounsaturated fatty acid.

Values are mean ± SD

^a^ p-value obtained based on Kruskal–Wallis test.
