# Supplementary material for: The relationship between a plant-based diet and mental health: Evidence from a cross-sectional multicentric community trial (LIPOKAP study)
Source: PLoS One. 2023 May 31;18(5):e0284446. doi: 10.1371/journal.pone.0284446 (PMC10231825; doi:10.1371/journal.pone.0284446)
Supplement: S3 Table — (DOCX) [file pone.0284446.s003.docx]

**Supplementary Table 3** Crude and multivariable-adjusted odds ratios and 95% CIs for anxiety and depression across quartiles of PDI, hPDI, and uPDI scores stratified by sex.

|  | PDI | | | |  | hPDI | | | |  | uPDI | | | |  |
| --- | --- | --- | --- | --- | --- | --- | --- | --- | --- | --- | --- | --- | --- | --- | --- |
|  | **Q1** | **Q2** | **Q3** | **Q4** | **P trend** | **Q1** | **Q2** | **Q3** | **Q4** | **P trend** | **Q1** | **Q2** | **Q3** | **Q4** | **P**  **trend** |
| Males |  |  |  |  |  |  |  |  |  |  |  |  |  |  |  |
| **Depression** |  |  |  |  |  |  |  |  |  |  |  |  |  |  |  |
| **Model I** | 1 | 0.84 (0.47, 1.48) | 0.97 (0.57, 1.63) | 1.07 (0.63, 1.82) | 0.648 | 1 | 0.78 (0.48, 1.26) | 0.60 (0.35, 1.02) | 0.94 (0.55, 1.60) | 0.418 | 1 | 1.33 (0.75, 2.35) | 2.19 (1.27, 3.76) | 1.96 (1.13, 3.40) | 0.004 |
| **Model II** | 1 | 0.80 (0.45, 1.43) | 0.95 (0.54, 1.66) | 0.98 (0.52, 1.84) | 0.643 | 1 | 0.72 (0.44, 1.19) | 0.53 (0.29, 0.94) | 0.82 (0.44, 1.51) | 0.305 | 1 | 1.42 (0.79, 2.56) | 2.36 (1.32, 4.21) | 2.17 (1.16, 4.06) | 0.003 |
| **Anxiety** |  |  |  |  |  |  |  |  |  |  |  |  |  |  |  |
| **Model I** | 1 | 0.66 (0.38, 1.15) | 0.72 (0.43, 1.21) | 0.82 (0.49, 1.39) | 0.590 | 1 | 1.25 (0.77, 2.03) | 1.19 (0.71, 1.98) | 1.05 (0.59, 1.86) | 0.792 | 1 | 1.56 (0.88, 2.75) | 2.50 (1.46, 4.31) | 1.66 (0.93, 2.95) | 0.024 |
| **Model II** | 1 | 0.63 (0.36, 1.12) | 0.72 (0.41, 1.24) | 0.74 (0.39, 1.37) | 0.147 | 1 | 1.20 (0.72, 2.00) | 1.09 (0.62, 1.89) | 0.88 (0.46, 1.70) | 0.866 | 1 | 1.64 (0.91, 2.94) | 2.63 (1.48, 4.68) | 1.74 (0.91, 3.31) | 0.026 |
| **Females** | 1 | 0.91 (0.66, 1.25) | 0.86 (0.62, 1.19) | 1.09 (0.76, 1.57) | 0.777 | 1 | 1.26 (0.91, 1.76) | 1.14 (0.81. 1.62) | 1.16 (0.81, 1.66) | 0.604 | 1 | 1.38 (0.98, 1.94) | 1.82 (1.30, 2.54) | 1.53 (1.07, 2.19) | 0.008 |
| **Depression** |  |  |  |  |  |  |  |  |  |  |  |  |  |  |  |
| **Model I** | 1 | 0.72 (0.48,1.07) | 0.92 (0.63, 1.34) | 0.92 (0.62, 1.36) | 0.916 | 1 | 1.30 (0.84, 2.02) | 1.40 (0.91, 2.15) | 1.39 (0.92, 2.10) | 0.137 | 1 | 1.54 (1.00, 2.37) | 1.54 (1.01, 2.36) | 1.98 (1.31, 2.99) | 0.002 |
| **Model II** | 1 | 0.74 (0.49, 1.12) | 1.03 (0.68, 1.55) | 1.09 (0.67, 1.75) | 0.948 | 1 | 1.15 (0.71, 1.83) | 1.15 (0.72, 1.85) | 1.05 (0.65, 1.69) | 0.172 | 1 | 1.75 (1.10, 2.77) | 1.54 (0.97, 2.45) | 1.88 (1.17, 3.03) | <0.0001 |
| **Anxiety** |  |  |  |  |  |  |  |  |  |  |  |  |  |  |  |
| **Model I** | 1 | 1.08 (0.74, 1.57) | 0.92 (0.63, 1.34) | 1.39 (0.96, 2.01) | 0.177 | 1 | 1.26 (0.84, 1.91) | 1.15 (0.77, 1.73) | 1.28 (0.87, 1.89) | 0.308 | 1 | 1.15 (0.77, 1.71) | 1.38 (0.94, 2.04) | 1.37 (0.93, 2.01) | 0.067 |
| **Model II** | 1 | 1.06 (0.72, 1.58) | 0.90 (0.60, 1.36) | 1.33 (0.84, 2.08) | <0.0001 | 1 | 1.32 (0.85, 2.06) | 1.18 (0.75, 1.85) | 1.27 (0.81, 1.99) | 0.386 | 1 | 1.30 (0.85, 1.98) | 1.50 (0.99, 2.28) | 1.43 (0.92, 2.22) | 0.060 |

PDI, overall plant-based diet index; hPDI, healthful plant-based diet index; uPDI, unhealthful plant-based diet index.

Model I: Crude model.

Model II: Additionally adjusted for age, sex, energy intake, marital status, education, physical activity level, and smoking.
